# Complete chloroplast genome sequence of *Caryocar brasiliense* Camb. (Caryocaraceae) and comparative analysis brings new insights into the plastome evolution of Malpighiales

**DOI:** 10.1590/1678-4685-GMB-2019-0161

**Published:** 2020-05-29

**Authors:** Rhewter Nunes, Ueric José Borges de Souza, Cintia Pelegrineti Targueta, Rafael Barbosa Pinto, Thannya Nascimento Soares, José Alexandre Felizola Diniz-Filho, Mariana Pires de Campos Telles

**Affiliations:** 1 Universidade Federal de Goiás (UFG), Instituto de Ciências Biológicas, Laboratório de Genética e Biodiversidade (LGBio), Goiânia, GO, Brazil; 2 Universidade Federal de Goiás (UFG), Instituto de Ciências Biológicas, Laboratório de Ecologia Teórica e Síntese (LETS), Goiânia, GO, Brazil; 3 Pontifícia Universidade Católica (PUC - GO), Escola de Ciências Agrárias e Biológicas, Goiânia, GO, Brazil

**Keywords:** Cerrado, genomics, molecular evolution, organellar genome, genome evolution

## Abstract

*Caryocar brasiliense* (Caryocaraceae) is a Neotropical tree species widely distributed in Brazilian Savannas. This species is very popular in central Brazil mainly by the use of its fruits in the local cuisine, and indeed it is one of the candidates, among Brazilian native plants, for fast track incorporation into cropping systems. Here we sequenced the complete chloroplast genome of *C. brasiliense* and used the data to access its genomic resources using high-throughput sequencing. The chloroplast exhibits a genome length of 165,793 bp and the typical angiosperm quadripartite structure with two copies of an inverted repeat sequence (IRa and IRb) of 34,902 bp each, separating a small single copy (SSC) region of 11,852 bp and a large single copy (LSC) region of 84,137 bp. The annotation analysis identified 136 genes being 87 protein-coding, eight rRNA and 37 tRNA genes. We identified 49 repetitive DNA elements and 85 microsatellites. A bayesian phylogenetic analysis helped to understand previously unresolved relationships in Malpighiales, placing Caryocaraceae as a separated group in the order, with high supported nodes. This study synthetizes valuable information for further studies allowing a better understanding of evolutionary patterns in the group and providing resources for future breeding programs.

In the order, Malpighiales, the family Caryocaraceae has not yet been explored using genomic approaches. Caryocaraceae represents a poorly resolved group within Malpighiales, forming a polytomy with other families, such as Malpighiaceae and Chrysobalanaceae, for which we have fully sequenced chloroplast genomes at the species level (Xi *et al.*, 2012; Angiosperm Phylogeny Group *et al.*, 2016). The main representatives of the Caryocaraceae family are the species of the genus *Caryocar* L., in particular *Caryocar brasiliense* Camb. This species is a Neotropical tree, the fruit of which is highly valued in Brazilian cuisine, a nutritious source for bats (Gribel and Hay, 1993), and is widely known in folk culture as a symbol of Brazilian savannas (or Cerrado) (Gribel and Hay, 1993; Araujo, 1995). The fruit pulp of *C. brasiliense* is rich in unsaturated fatty acids, vitamins, and phenolic acids, as well in carotenoids, such as violaxanthin, lutein, and zeaxanthin (Castro *et al.*, 2008; Mariano *et al.*, 2009). Because of all these characteristics, *C. brasiliense* is one of the main native Cerrado species that is a candidate for incorporation into cropping systems (Leite *et al.*, 2006; Tunholi *et al.*, 2013).

Despite the importance of *Caryocar brasiliense*, until now no genomic resources have been developed for this species. A few studies have used microsatellite markers or short sequences to evaluate the genetic diversity patterns of this species, showing that the natural populations of *C. brasiliense* have a relatively low genetic structure and a high genetic and phylogeographic diversity (e.g. Diniz-Filho *et al.*, 2009; Collevatti *et al.*, 2011). Thus, in this study, we sequenced the complete chloroplast genome of *C. brasiliense* and used the data to access its genomic resources using high-throughput sequencing. As a result, we generated information regarding the chloroplast genome sequence, gene composition and organization, and repeat sequences using part of our data to reconstruct a phylogenetic tree for the Malpighiales order to analyze the relationships of *C. brasiliense* within this group.

The total DNA of the fresh leaves of *Caryocar brasiliense* was extracted using the CTAB protocol (Doyle and Doyle, 1987). The sample was sequenced using an Illumina HiSeq2000 platform in paired-end 2100 bp mode. Raw reads were evaluated for base quality sequencing and the presence of sequencing adapters using FastQC software (Andrews, 2010). Quality control was performed using Trimmomatic (Bolger *et al.*, 2014) software. The resulting high-quality reads were selected for *de novo* chloroplast genome assembly using NOVOPlasty v.2.7.1 software (Dierckxsens *et al.*, 2017). We performed gene annotation of the *Caryocar brasiliense* chloroplast genome using CHLOROBOX GeSeq (Tillich *et al.*, 2017) and Dual Organellar GenoMe Annotator (DOGMA) (Wyman *et al.*, 2004) software. Simple sequence repeats (SSR) or microsatellite regions were predicted in the *C. brasiliense* chloroplast genome using Imperfect Microsatellite Extractor (IMEx) (Mudunuri and Nagarajaram, 2007). Repeat sequence elements were predicted using REPuter software (Kurtz, 2002). In addition, we performed a Bayesian phylogenetic analysis for several Malpighiales species using 76 protein-coding gene sequences. For a detailed description of the methods, please refer to the Supplementary Material.

The complete sequence of the *Caryocar brasiliense* chloroplast genome was deposited in the GenBank database (accession number: MK726375) with a high mean genome coverage (715X). The plastome of *C. brasiliense* exhibited a total length of 165,793 bp and typical quadripartite division, which has previously been observed in other flowering plants (Figure 1; Figure S1). The genome was comprised of a large single copy (LSC), a small single copy (SSC), and a pair of inverted repeats (IRa and IRb). These regions were comprised of 84,137 bp, 11,852 bp, and 34,902 bp, respectively, with a GC content of 36.7%. The IR regions exhibited the greatest GC content (39.6%), followed by LSC (35.0%) and SSC (31.5%). Within the inverted repeat regions, the GC content was higher where the rRNAs were predicted.

**Figure 1:**
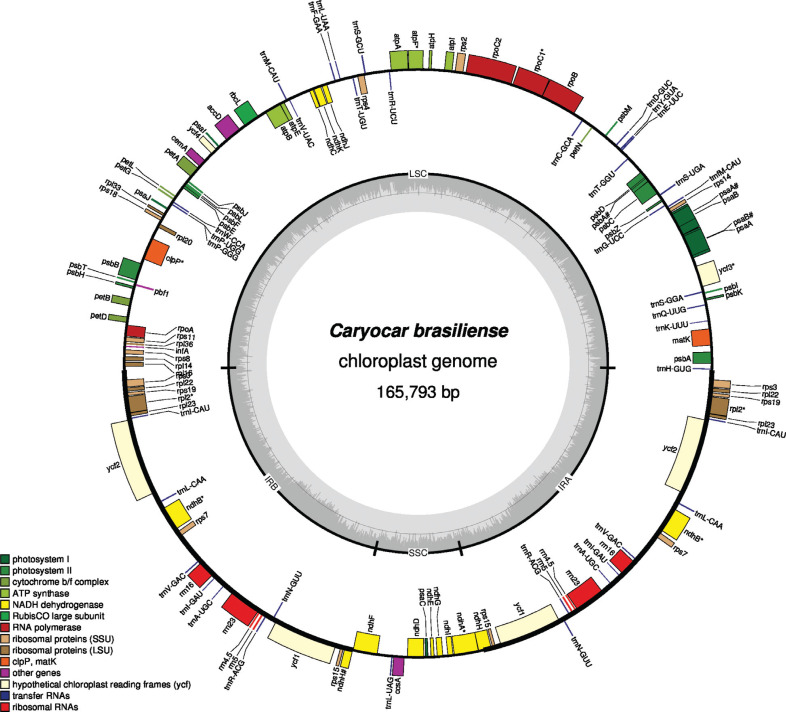
Chloroplast genome map of *Caryocar brasiliense*. The genes drawn outside and inside of the circle are transcribed in clockwise and counterclockwise directions, respectively. Genes were colored based on their functional groups. The inner circle shows the quadripartite structure of the chloroplast: small single copy (SSC), large single copy (LSC) and a pair of inverted repeats (IRa and IRb). The gray ring marks the GC content with the inner circle marking a 50% threshold. Genes that have introns were marked with “*” and pseudogenes were marked with “#”.

We compared the structural features of the *C. brasiliense* chloroplast genome with another nine chloroplast genomes from nine other families in the Malpighiales order. In comparison, *C. brasiliense* had the largest genome size, with large IR regions, but one of the smallest small single copies regions, along with *Linum usitatissimum* L. (Souza *et al.*, 2017) (Table 1). However, *C. brasiliense* showed a similar size with respect to the LSC region of other species. This may be explained by the fact that many of the photosynthetic genes are present in this region, and are therefore important for the persistence of these species, resulting in fewer contraction/expansion events in the region of the chloroplast genome.

**Table 1:** Comparative chloroplast genome structural features in 10 species from Malpighiales order. LSC: Large Single Copy region; SSC: Small Single Copy region; IR: Inverted Repeats regions and bp: base pair.

Species	Family	Genome size (bp)	LSC (bp)	SSC (bp)	IR (bp)	GC(%)
*Caryocar brasiliense*	Caryocaraceae	165,793	84,137	11,852	34,902	36.7
*Garcinia mangostana*	Clusiaceae	158,179	86,458	17,703	27,009	36.1
*Chrysobalanus icaco*	Chrysobalanaceae	163,937	89,188	19,817	26,885	36.2
*Erythroxylum novogranatense*	Erythroxylaceae	163,937	91,384	18,137	27,208	35.9
*Manihot esculenta*	Euphorbiaceae	161,453	89,295	18,250	26,954	35.9
*Linum usitatissimum*	Linaceae	156,721	81,767	10,974	31,990	37.5
*Byrsonima coccolobifolia*	Malpighiaceae	160,329	88,524	17,833	26,986	36.8
*Passiflora edulis*	Passifloraceae	151,406	85,720	13,378	26,154	37.0
*Populus tremula*	Salicaceae	156,067	84,367	16,670	27,509	36.8
*Viola seoulensis*	Violaceae	156,507	85,691	18,008	26,404	36.3

We found 115 different genes in the genome, of which 77 were protein-coding genes, four ribosomal RNAs, 30 transfer RNAs, and four pseudogenes (Table S1). In addition, 10 protein-coding genes, four rRNAs, and seven tRNAs were found to be duplicated. Three of these tRNA genes, namely *trnA-UGC*, *trnM-CAU*, and *trnR-ACG*, were found to have more than one copy in the genome (each gene appeared four times). As such, taking into consideration these duplicated genes, the *C. brasiliense* chloroplast genome had a total of 136 genes (87 protein-coding genes, four pseudogenes, eight rRNAs, and 37 tRNAs). We also observed 10 genes that contained introns. Over all, *C. brasiliense* was found to have a relatively conserved number of genes compared to the other Malpighiales species, especially in terms of the genes related to rRNA, tRNA, and photosynthesis. The gene features of *C. brasiliense* were very similar to those of *Byrsonima coccolobifolia* Kunth (Menezes *et al.*, 2018), another Malpighiales species (Table S2).

The pseudogenes predicted in the *C. brasiliense* chloroplast genome were *ndhH*, *psaA*, *psaB*, and *psbA*. These pseudogenes had a copy of the gene in the complete form and are supposedly involved in the structural changes of the chloroplast genome in *C. brasiliense*. For example, the *ndhH* pseudogene was related to the process of duplication of the Inverted Repeat region, while the others pseudogenes were related to the inversion events in the Large Single Copy region, generating variation in the order of the plastid genes in comparison to the chloroplast genomes of other Malpighiales species. Such inversion events were also observed in the chloroplast genome of *Passiflora edulis* Sims, another Malpighiales species (Cauz-Santos *et al.*, 2017), which is indicative of a non-conservative gene collinearity found in all members of this order.

A comparative analysis with 10 Malpighiales species revealed a highly conserved pattern of variation within the genomes (highly variable regions between *C. brasiliense* and *Jatropha curcas* were also very variable in other species compared to *J. curcas*), with highly conserved protein-coding genes, as well as intergenic regions with more variation (Figure S2). *Passiflora edulis* had the highest divergent regions in comparison to *Jatropha curcas* L. and other species, with a similarity between regions of less than 50%.

Compared to other species under analysis, *Caryocar brasiliense* displayed one of the smallest Small Single Copy (SSC) regions and the largest Inverted Repeat (IR) regions. This indicates that the size of these regions evolves differently between different species of Malpighiales (Mower *et al.*, 2015). We performed an IR boundaries comparison analysis to determine the genes present at the sites that separate the chloroplast regions (Figure 2). The most common gene to flank the region between the IR and SSC was *ycf1* in seven of the ten species analyzed. Commonly, *ycf1* and *ycf1* pseudogene were present in the IR/SSC boundaries in Malpighiales species, such as *Byrsonima coccolobifolia* (Menezes *et al.*, 2018), *Chrysobalanus icaco* L. (Bardon *et al.*, 2016), *Erythroxylum novogranatense* (D. Morris) Hieron. (unpublished), *Garcinia mangostana* L. (Jo *et al.*, 2017), *Manihot esculenta* Crantz (Daniell *et al.*, 2008), *Populus tremula* L. (Kersten *et al.*, 2016), and *Viola seoulensis* Nakai (Cheon *et al.*, 2017).

**Figure 2:**
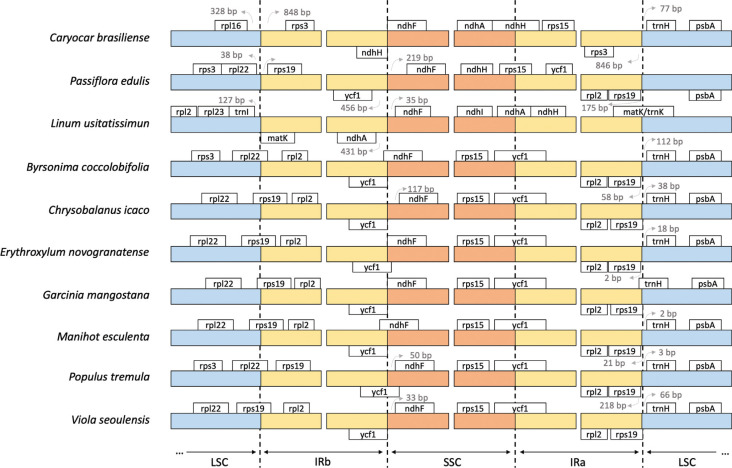
Comparison of the junctions involving Inverted Repeat (IRa and IRb) regions with Large Single Copy (LSC) and Small Single Copy (SSC) regions among 10 chloroplast genomes of Malpighiales. The IR regions are represented in yellow whereas LSC and SSC in blue and orange, respectively. The white boxes represent the genes present in each region. The arrows represent the distance (in base pairs) of genes from the junction site between regions.

Three of the species which were subjected to analysis did not have *ycf1* as a flanking gene of the IR/SSC region, *Caryocar brasiliense*, *Passiflora edulis* (Cauz-Santos *et al.*, 2017), and *Linum usitatissimum* L. (Souza *et al.*, 2017). *Caryocar brasiliense* had a boundary between the IR/SSC region, flanked by *ndhH* and *ndhH* pseudogene, while in *P. edulis*, *rps15* and intergenic region *ycf1-ndhF* were found. For *L. usitatissimum, ndhA* and the intergenic region *ndhA-ndhF* were observed. The IR/SSC boundary region was found to have a high collinearity between different genomes. The order of the conserved genes was as follows: *ndhA*, *ndhH*, *rps15*, and *ycf1* for all species under analysis. Meanwhile, the bounder of IR/SSC for the major species included *ycf1*, however, in some Malpighiales species, this site was displaced with the donation of genomic segments from the SSC region to the IR regions. In addition to colinear genetic evidence, this pattern of expansion of the IR regions with the contraction of the SSC region was also supported by the region lengths, which indicated that species with no *ycf1* flanking the IR/SSC boundery had a smaller SSC region (Table S2).

A total of 85 perfect microsatellites (SSRs) were identified in the *Caryocar brasiliense* chloroplast genome sequence (Figure S3). With respect to the repeat motif, we found 52 mononucleotides, 11 dinucleotides, five trinucleotides, 12 tetranucleotides, three pentanucleotides, and two hexanucleotides. The number of continuous repeats (motif iterations) ranged from 3 to 16 (Table S3). The chloroplast region with the highest number of SSRs were LSC (56.47%), followed by IR (29.41%) and SSC (14.12%). SSRs are important regions because they serve as molecular markers in studies on genetic diversity, phylogeny, and phylogeography for Brazilian Savanna species (Rabelo *et al.*, 2011; Soares *et al.*, 2012; Telles *et al.*, 2013). The SSR regions identified can be used in molecular marker development testing for genetic diversity studies of *C. brasiliense* and related species (Table S4).

We also identified repeat sequences in the *C. brasiliense* chloroplast genome and another 10 species from the Malpighiales order (chloroplast genome subset described in Material and Methods) (Table S5). We observed a total of 49 repeats in *C. brasiliense*. With regards to the type of repeat, we found 18 forward, 30 palindromic, and one reverse repeat. No repeats of the complement type were observed in *C. brasiliense*. Complement repeats only occurred in three of the 10 species in analysis: one in *Chrysobalanus icaco*, two in *Erythroxylum novogranatense*, and one in *Viola seoulensis*. In another three species, *Linum usitatissimum*, *Manihot esculenta*, and *Passiflora edulis*, we only found forward and palindromic repeats. Repeat analysis revealed a highly conserved total number of repeats within the Malpighiales order, although the type of repeats varied across species.

We performed a phylogenetic analysis by sampling 52 representatives from all the families in the Malpighiales order with sequenced chloroplast genomes using protein-coding gene sequences (Table S6). Additionally, we also retrieved chloroplast gene sequences from *Anthodiscus peruanus* Baill. (Caryocaraceae) and *Putranjiva roxburghii* Wall. (Putranjivaceae). This analysis resulted in a phylogenetic tree with high supported values for the nodes, with a Bayesian posterior probability given to each one (Figure 3). As expected, all the species analyzed (including *C. brasiliense*) fell within the clades that represent their respective botanical families, validating the chloroplast sequences obtained in this study.

**Figure 3:**
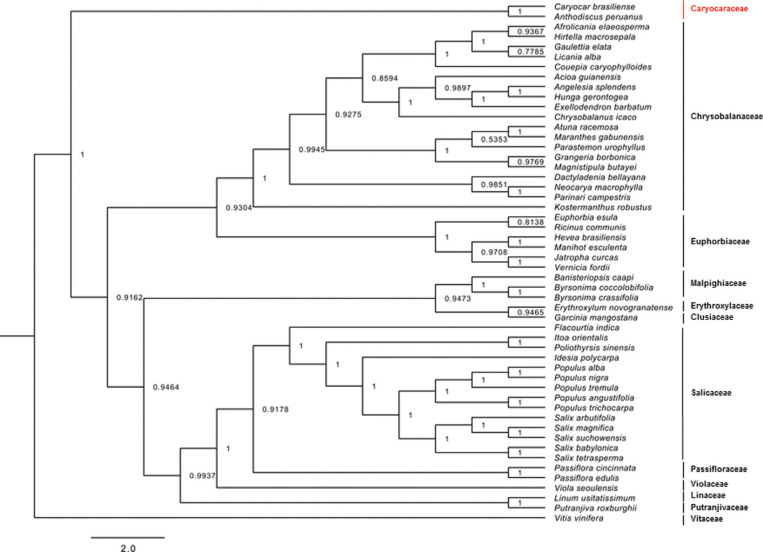
Phylogenetic tree reconstruction based on 52 taxa using Bayesian inference based on 76 protein-coding chloroplast genes. Numbers represent the Bayesian posterior probability given to each node. The bars on the right represents the botanic families of species.

The currently accepted phylogeny for the order Malpighiales displays a polytomy involving Caryocaraceae, Putranjivoid, Malpighioid, and Chrysobalanoid species (Angiosperm Phylogeny Group *et al.*, 2003; Xi *et al.*, 2012). Here, for the first time, a highly supported phylogenetic tree identified Caryocaraceae as a sister clade in relation to the other families within Malpighiales (Figure 3). Moreover, this result provides evidence that reinforces the non-clustering among Caryocaraceae, Linaceae, and Erythroxylaceae within the same clade, as discussed in a previous study (Soltis *et al.*, 2007). The current phylogeny of this group was investigated using several genes. The novel genomic resources produced in this study will help to improve our understanding of the phylogenetic relationships among species in the order Malpighiales (Angiosperm Phylogeny Group *et al.*, 2003; Angiosperm Phylogeny Group *et al.*, 2009; Xi *et al.*, 2012; Angiosperm Phylogeny Group *et al.*, 2016). These resources will help to resolve the problem of uncertain clades. This study demonstrates how the use of high-throughput sequencing technologies can increase the accuracy of phylogenetic analysis. Moreover, the data provided here serve as a novel genetic and genomic resource for Malpighiales and offer the first complete genome sequence and chloroplast content in the Caryocaraceae family.

## Supplementary Material

The following online material is available for this article:

Figure S1 Distribution of k-mers in *Caryocar brasiliense* chloroplast genome.

Figure S2 Alignment view of chloroplast genomes of Malpighiales order using *Jatropha curcas* (Euphorbiaceae) as reference.

Figure S3 Repeat and comparative analysis in *Caryocar brasiliense* chloroplast genome.

Table S1 Gene content and classification of *Caryocar brasiliense* chloroplast genome.

Table S2 Comparative chloroplast genome gene features in 10 species from Malpighiales order.

Table S3 Frequency of types of simple sequence repeats based on its motif length in *Caryocar brasiliense* chloroplast genome.

Table S4 Simple sequence repeats identified in *Caryocar brasiliense* chloroplast genome sequence.

Table S5 Species list used in comparative analysis of Malpighiales order chloroplast genomes.

Table S6 Species list used in phylogenetic analysis of Malpighiales order.

Method details.
