# Supplementary material for: Complete chloroplast genome sequence of Caryocar brasiliense Camb. (Caryocaraceae) and comparative analysis brings new insights into the plastome evolution of Malpighiales
Source: Genet Mol Biol. 2020 May 29;43(2):e20190161. doi: 10.1590/1678-4685-GMB-2019-0161 (PMC7263422; doi:10.1590/1678-4685-GMB-2019-0161)
Supplement: Supplementary file 1 [file 1415-4757-GMB-43-2-e20190161-s10.pdf]

Genetics and Molecular Biology 43, 2 (2020)

Supplementary Material to "Complete chloroplast genome sequence of *Caryocar brasiliense* Camb. (Caryocaraceae) and comparative analysis brings new insights into the plastome evolution of Malpighiales"

1. Method details

1.1 DNA sampling and sequencing

Fresh leaves were collected from a tree at Escola de Agronomia, Universidade Federal de Goiás, Goiânia, Goiás, Brazil. The total DNA was extracted from leaves using CTAB protocol. The quality of DNA was measured by Nanodrop and the quantity by Qubit and agarose gel. The sample was sent to Centro de Genômica Funcional ESALQ-USP core-facility for sequencing. An Illumina paired-end 2x100 bp library was constructed and forwarded for sequencing in an Illumina HiSeq2000 platform.

1.2 Sequencing quality control, assembly and validation

Raw reads were evaluated for base quality sequencing and sequencing adapters presence using FastQC software (Andrews, 2010). The quality control was performed using Trimmomatic (Bolger *et al.*, 2014) software with the options ILLUMINACLIP: TruSeq3-PE.fa:2:30:10 and SLIDEWINDOW: 4:30, that is, at least a mean phred-score of 30 every four bases. The high-quality reads were taken for a *de novo* chloroplast genome assembly in NOVOPlasty v.2.7.1 software (Dierckxsens *et al.,* 2017). The *Caryocar brasiliense psbA* gene sequence (GenBank: EU350266.1) was used as seed to extend the chloroplast genome sequence in assembly analysis. The assembled chloroplast genome and the junctions between inverted repeats and single copy regions were validated in a k-mer coverage analysis using Jellyfish software (Marçais and Kingsford, 2011) (Figure S1).

1.3 Chloroplast structure and gene annotation

We performed a gene annotation of the *Caryocar brasiliense* chloroplast genome using CHLOROBOX GeSeq (Tillich *et al.,* 2017) and DOGMA - Dual Organellar GenoMe Annotator (Wyman *et al.,* 2004) software.

Predicted genes were curated using BLAT and BLAST searches in a database with chloroplastidial Embryophyta CDS and RNA reference sequences. ARAGORN v1.2.38 (Laslett and Canback, 2004) and tRNAscan-SE v2.0 (Lowe and Eddy, 1997; Lowe and Chan, 2016) softwares were also used to predict tRNA sequences. ARAGORN was configured in "Bacterial/Plant plastid" genetic code mode and with a maximum intron length of 3000 bp. tRNAscan-SE was configured in "Organellar tRNAs" and a cut-off score for reporting tRNAs of 15. The circular chloroplast genome map was created using OrganellarGenomeDRAW (Lohse *et al.*, 2013).

1.4 Comparative analysis among Malpighiales species

Comparative analysis of structure and composition of chloroplast genome sequences were performed using a dataset compounded by nine species in Malpighiales order: *Byrsonima coccolobifolia* (NC_037191.1), *Chrysobalanus icaco* (NC_024061.1), *Erythroxylum novogranatense* (NC_030601.1), *Garcinia mangostana* (NC_036341.1), *Linum usitatissimum* (NC_036356.1), *Manihot esculenta* (NC_010433.1), *Passiflora edulis* (NC_034285.1), *Populus tremula* (NC_027425.1) and *Viola seoulensis* (NC_026986.1). The species dataset represents all families in Malpighiales with chloroplast genomes sequenced until now. The plastid genomes were retrieved from GenBank, as well as its genome annotation information. The dataset was used in whole-genome alignment view, repeat sequence and IR boundaries analysis. Comparisons were performed using Geneious software (Duran *et al.*, 2012).

An alignment view of chloroplast genomes of Malpighiales were drawn using mVISTA software (Frazer *et al.*, 2004). *Jatropha curcas* (Euphorbiaceae) (NC_012224.1) chloroplast genome was used as reference (Asif *et al.*, 2010). The analysis was conducted in Shuffle-LAGAN mode (Global pair-wise alignment of finished sequences) and probability threshold of 0.5. To facilitate the visualization, a resumed alignment figure was made using only five species (Cbr: *Caryocar brasiliense*; Cic: *Chrysobalanus icaco*; Bco: *Byrsonima coccolobifolia*; Mes: *Manihot esculenta*; Ped: *Passiflora edulis*).

1.5 Repeat sequence analysis

Simple sequence repeats (SSR) or microsatellite regions were predicted in *C. brasiliense* chloroplast genome using IMEx - Imperfect Microsatellite Extractor (Mudunuri and Nagarajaram, 2007). We used the following minimum repeat number criteria: ten units for mononucleotide, five units for dinucleotide, four units for trinucleotide and three units for tetra, penta and hexanucleotides, respectively. All repeat sequence positions were retrieved using Geneious software (Duran *et al.*, 2012).

We looked for repeat sequence elements in *Caryocar brasiliense* chloroplast genome using REPuter software (Kurtz, 2002). Forward, reverse, complement and palindromic repeats types were searched using a minimal repeat size of 30 bp and a Hamming distance of 3 (so, sequence identities ≥ 90%). We used the same search criteria to identify repeat sequence elements in the chloroplast genomes of *Byrsonima coccolobifolia, Chrysobalanus icaco*, *Erythroxylum novogranatense*, *Garcinia mangostana, Linum usitatissimum, Manihot esculenta, Passiflora edulis, Populus tremula* and *Viola seoulensis.* We used this information in a quantitative comparative analysis among families from Malpighiales order.

1.6 Phylogenetic analyses

We performed a Bayesian phylogenetic analysis among some Malpighiales species using 76 protein-coding gene sequences. We retrieved coding sequences (CDS) from 49 complete chloroplast genome sequences from Nucleotide NCBI (National Center for Biotechnology Information) database representing all the families in Malpighiales order that had their chloroplast genomes sequence until now: Chrysobalanaceae, Clusiaceae, Erythroxylaceae, Euphorbiaceae, Linaceae, Malpighiaceae, Passifloraceae, Salicaceae e Violaceae. Additionally, we also collected chloroplast gene sequences from *Anthodiscus peruanus* (Caryocaraceae) and *Putranjiva roxburghii* (Putranjivaceae) (Xi *et al.*, 2012). *Vitis vinifera* L. (Vitaceae) chloroplast gene sequences were used as outgroup. The GenBank accessions are listed in supplement material (Table S6).

The 76 shared protein-coding genes were aligned separately by gene using MAFFT v. 7 (Katoh and Standley, 2013) server software. Alignment files were concatenated in a single matrix using Sequence Matrix software (Vaidya *et al.*, 2011). Informative sites were extracted from matrix using GBlocks v. 0.91b (Cruickshank, 2000) and was used as input in jModelTest v. 2.1.10 (Posada, 2008; Darriba *et al.*, 2012) to test

the best-fitting evolution model for phylogeny estimation based on the Akaike Information Criterion (AIC). The model GTR+G+I was chose like evolutionary model in phylogenetic analysis using a Bayesian inference approach in MrBayes v. 3.2 software (Huelsenbeck and Ronquist, 2001; Darling *et al.*, 2012). This analysis was conducted using two independent Markov chain Monte Carlo (MCMC) chains with three hot chains and one cold chain each and 2 x 5,000,000 generations. Phylogenetic trees were sampled every 1,000 generations and the first 25% were discarded as burn-in. The remaining trees were used to construct majority-rule consensus. The MCMC convergence was assumed when the average standard deviation of split frequencies reached 0.01 or less. The runs were evaluated using Tracer software (Rambaut *et al.*, 2018). Phylogenetic tree visualization was drawn using FigTree (Rambaut, 2010).

References

Andrews S (2010) FastQC: a quality control tool for high throughput sequence data, http://www.bioinformatics.babraham.ac.uk/projects/fastqc.

Asif MH, Mantri SS, Sharma A, Srivastava A, Trivedi I, Gupta P, Mohanty CS, Sawant SV and Tuli R (2010) Complete sequence and organisation of the Jatropha curcas (Euphorbiaceae) chloroplast genome. Tree Genet Genomes 6:941-952.

Bolger AM, Lohse M and Usadel B (2014) Trimmomatic: A flexible trimmer for Illumina sequence data. Bioinformatics 30:2114-2120.

Cruickshank R (2000) Selection of conserved blocks from multiple alignments for their use in phylogenetic analysis. Mol Biol Evol 17:540-52.

Darling A, Ronquist F, Ayres DL, Larget B, Liu L, Teslenko M, Suchard MA, Huelsenbeck JP, Höhna S and van der Mark P (2012) MrBayes 3.2: Efficient Bayesian Phylogenetic Inference and Model Choice Across a Large Model Space. Syst Biol 61:539-542.

Darriba D, Taboada GL, Doallo R and Posada D (2012) JModelTest 2: More models, new heuristics and parallel computing. Nat Methods 9:772.

Dierckxsens N, Mardulyn P and Smits G (2017) NOVOPlasty: De novo assembly of organelle genomes from whole genome data. Nucleic Acids Res 45:e18.

Duran C, Markowitz S, Moir R, Cooper A, Ashton B, Drummond A, Buxton S, Sturrock S, Wilson A, Thierer T *et al.* (2012) Geneious Basic: An integrated and extendable desktop software platform for the organization and analysis of sequence data. Bioinformatics 28:1647-1649.

Frazer KA, Pachter L, Poliakov A, Rubin EM and Dubchak I (2004) VISTA: Computational tools for comparative genomics. Nucleic Acids Res 32:273-279.

Huelsenbeck JP and Ronquist F (2001) MrBAYES; Bayesian inference for phylogeny. Bioinformatics 17:754-755.

Katoh K and Standley DM (2013) MAFFT multiple sequence alignment software version 7: Improvements in performance and usability. Mol Biol Evol 30:772-780.

Kurtz S (2002) REPuter: the manifold applications of repeat analysis on a genomic scale. Nucleic Acids Res 29:4633-4642.

Laslett D and Canback B (2004) ARAGORN, a program to detect tRNA genes and tmRNA genes in nucleotide sequences. Nucleic Acids Res 32:11-16.

Lohse M, Drechsel O, Kahlau S and Bock R (2013) OrganellarGenomeDRAW--a suite of tools for generating physical maps of plastid and mitochondrial genomes and visualizing expression data sets. Nucleic Acids Res 41:575-581.

Lowe TM and Chan PP (2016) tRNAscan-SE On-line: integrating search and context for analysis of transfer

RNA genes. Nucleic Acids Res 44:W54-W57.

Lowe TM and Eddy SR (1997) tRNAscan-SE: A Program for Improved Detection of Transfer RNA Genes in Genomic Sequence. Nucleic Acids Res 25:0955-0964.

Marçais G and Kingsford C (2011) A fast, lock-free approach for efficient parallel counting of occurrences of k-mers. Bioinformatics 27:764-770.

Mudunuri SB and Nagarajaram HA (2007) IMEx: Imperfect microsatellite extractor. Bioinformatics 23:1181-1187.

Posada D (2008) jModelTest: Phylogenetic model averaging. Mol Biol Evol 25:1253-1256.

Rambaut A, Drummond AJ, Xie D, Baele G and Suchard MA (2018) Posterior Summarization in Bayesian Phylogenetics Using Tracer 1.7. Syst Biol 67:901-904.

Rambaut A (2010) FigTree v1.3.1, http://tree.bio.ed.ac.uk/software/figtree/.

Tillich M, Lehwark P, Pellizzer T, Ulbricht-Jones ES, Fischer A, Bock R and Greiner S (2017) GeSeq - Versatile and accurate annotation of organelle genomes. Nucleic Acids Res 45:W6-W11.

Vaidya G, Lohman DJ and Meier R (2011) SequenceMatrix: Concatenation software for the fast assembly of multi-gene datasets with character set and codon information. Cladistics 27:171-180.

Wyman SK, Jansen RK and Boore JL (2004) Automatic annotation of organellar genomes with DOGMA. Bioinformatics 20:3252-3255.

Xi Z, Ruhfel BR, Schaefer H, Amorim AM, Sugumaran M, Wurdack KJ, Endress PK, Matthews ML, Stevens PF, Mathews S *et al.* (2012) Phylogenomics and a posteriori data partitioning resolve the Cretaceous angiosperm radiation Malpighiales. Proc Natl Acad Sci U S A 109:17519-17524.
