# Supplementary material for: Complete chloroplast genome sequence of Caryocar brasiliense Camb. (Caryocaraceae) and comparative analysis brings new insights into the plastome evolution of Malpighiales
Source: Genet Mol Biol. 2020 May 29;43(2):e20190161. doi: 10.1590/1678-4685-GMB-2019-0161 (PMC7263422; doi:10.1590/1678-4685-GMB-2019-0161)
Supplement: Supplementary file 3 [file 1415-4757-GMB-43-2-e20190161-s4.pdf]

Supplementary Material to "Complete chloroplast genome sequence of *Caryocar brasiliense* Camb. (Caryocaraceae) and comparative analysis brings new insights into the plastome evolution of Malpighiales"

Table S1 - Gene content and classification of *Caryocar brasiliense* chloroplast genome.

Gene category	Gene group	Gene name
Photosynthesis	Photosystem I	psaA, psaB, psaC, psaI, psaJ
	Photosystem II	psbA, psbB, psbC, psbD, psbE, psbF, psbH, psbI, psbJ, psbK, psbL, psbM, psbT, psbZ, pbf1
	Cytochrome b6/f complex	petA, petB, petD, petG, petL, petN
	ATP synthase	atpA, atpB, atpE, atpF[a], atpH, atpI
	Cytochrome c synthesis	ccsA
	Assembly/stability of photosystem I	ycf3[a], ycf4
	NADPH dehydrogenase	ndhA[a], ndhB[a,b], ndhC, ndhD, ndhE, ndhF, ndhG, ndhH, ndhI, ndhJ, ndhK
	Rubisco	rbcL
Transcription and translation	Transcription	rpoA, rpoB, rpoC1[a], rpoC2
	Ribosomal proteins	rpl2[a,b], rpl14, rpl16, rpl20, rpl22[b], rpl23[b], rpl33, rpl36, rps2, rps3[b], rps4, rps7[b], rps8, rps11, rps12[a], rps14, rps15[b], rps18, rps19[b]
Non-coding RNA genes	Ribosomal RNA	rrn4.5[b], rrn5[b], rrn16[b], rrn23[b]
	Transfer RNA	TrnA-UGC[a,b], trnC-GCA, trnD-GUC, trnE-UUC[a,b], trnF-GAA, trnfM-CAU, trnG-UCC, trnH-GUG, trnI-CAU, trnI-GAU[a,b], trnK-UUU, trnL-CAA[b], trnL-UAA, trnL-UAG, trnM-CAU[b], trnN-GUU, trnP-GGG, trnP-UGG, trnQ-UUG, trnR-ACG[b], trnR-UCU, trnS-GCU, trnS-GGA, trnS-UGA, trnT-GGU, trnT-UGU, trnV-GAC[b], trnV-UAC, trnW-CCA, trnY-GUA
Other genes	RNA processing	matK
	Carbon metabolism	cemA
	Fatty acid synthesis	accD
	Proteolysis	clpP[a]
	Translation initiation factor	InfA
	Component of TIC complex	ycf1[b]
Genes of unknown function	Conserved reading frames	ycf2[b]
Pseudogenes	-	ndhH, psaA, psaB, psbA

[a] Gene containing intron; [b] Gene located in inverted repeat regions;
