# Supplementary material for: Complete chloroplast genome sequence of Caryocar brasiliense Camb. (Caryocaraceae) and comparative analysis brings new insights into the plastome evolution of Malpighiales
Source: Genet Mol Biol. 2020 May 29;43(2):e20190161. doi: 10.1590/1678-4685-GMB-2019-0161 (PMC7263422; doi:10.1590/1678-4685-GMB-2019-0161)
Supplement: Supplementary file 4 [file 1415-4757-GMB-43-2-e20190161-s5.pdf]

Supplementary Material to "Complete chloroplast genome sequence of *Caryocar brasiliense* Camb. (Caryocaraceae) and comparative analysis brings new insights into the plastome evolution of Malpighiales"

Table S2 - Comparative chloroplast genome gene features in 10 species from Malpighiales order. rRNA: ribosomal RNA; tRNA: transfer RNA; CDS: coding sequences.

**Species**	**Family**	**Number of genes**	**rRNA**	**tRNA**	**CDS**	**Pseudogenes**
Caryocar brasiliense	Caryocaraceae	136	8	37	87	4
Garcinia mangostana	Clusiaceae	130	8	37	83	2
Chrysobalanus icaco	Chrysobalanaceae	130	8	37	83	2
Erythroxylum novogranatense	Erythroxylaceae	131	4	38	85	4
Manihot esculenta	Euphorbiaceae	131	4	38	83	6
Linum usitatissimum	Linaceae	133	8	37	83	5
Byrsonima coccolobifolia	Malpighiaceae	139	8	37	88	6
Passiflora edulis	Passifloraceae	133	8	36	76	13
Populus tremula	Salicaceae	123	8	29	85	1
Viola seoulensis	Violaceae	131	8	37	84	2
