# Supplementary material for: Complete chloroplast genome sequence of Caryocar brasiliense Camb. (Caryocaraceae) and comparative analysis brings new insights into the plastome evolution of Malpighiales
Source: Genet Mol Biol. 2020 May 29;43(2):e20190161. doi: 10.1590/1678-4685-GMB-2019-0161 (PMC7263422; doi:10.1590/1678-4685-GMB-2019-0161)
Supplement: Supplementary file 5 [file 1415-4757-GMB-43-2-e20190161-s2.pdf]

Supplementary Material to “Complete chloroplast genome sequence of *Caryocar brasiliense* Camb. (Caryocaraceae) and comparative analysis brings new insights into the plastome evolution of Malpighiales”

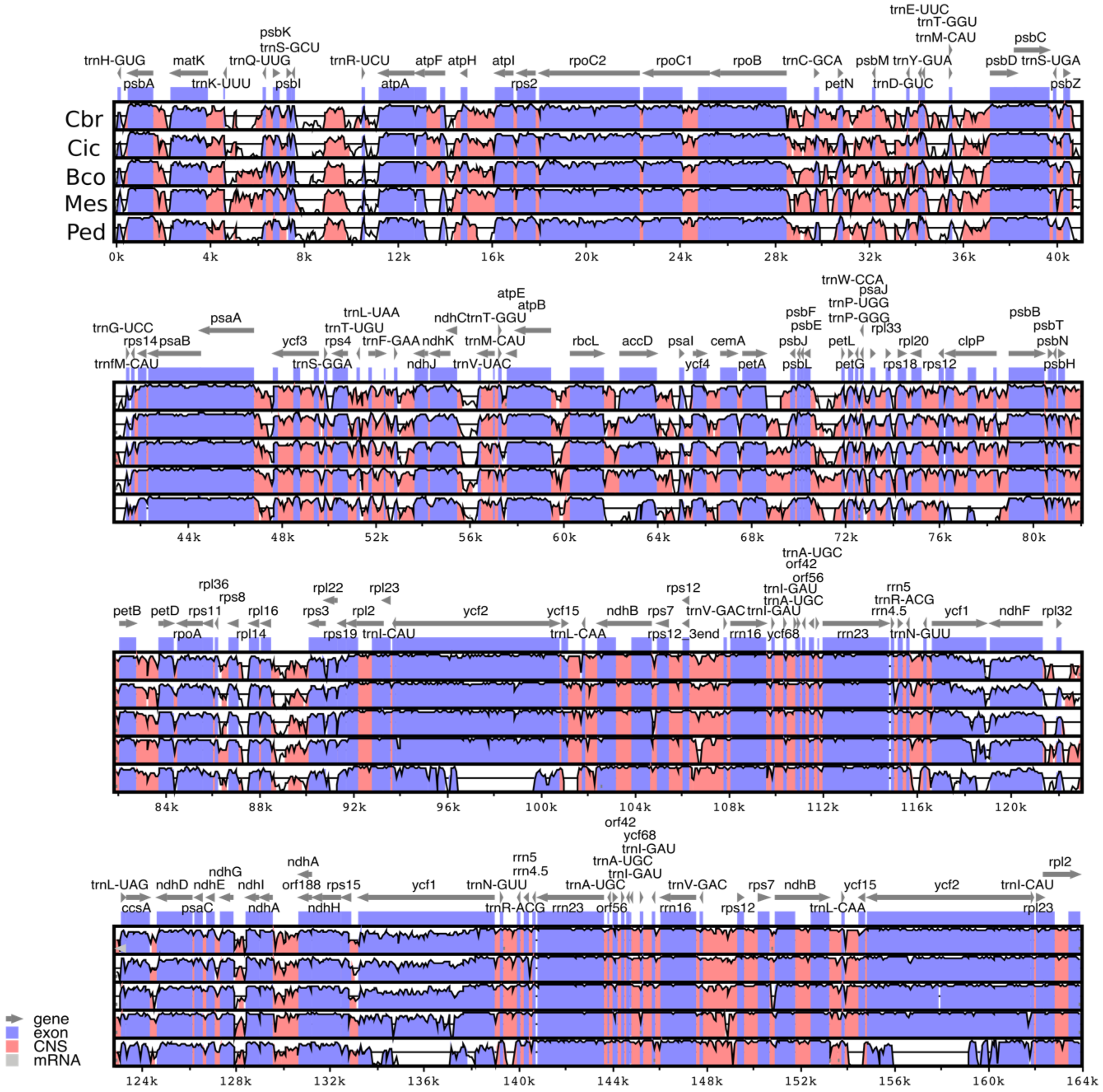

Figure S2 - Alignment view of chloroplast genomes of Malpighiales order using *Jatropha curcas* (Euphorbiaceae) as reference. This figure was draw using Mvista software. Grey arrows above the alignment indicates gene orientation. Pink regions represents CNS (Conserved Non-coding Regions). A threshold of 50% identity was used for the plots. Top and bottom of each horizontal bar represents a range of 50% to 100% of identity. Cbr: *Caryocar brasiliense*; Cic: *Chrysobalanus icaco*; Bco: *Byrsonima coccolobifolia*; Mes: *Manihot esculenta* and Ped: *Passiflora edulis*.
