# Supplementary material for: Complete chloroplast genome sequence of Caryocar brasiliense Camb. (Caryocaraceae) and comparative analysis brings new insights into the plastome evolution of Malpighiales
Source: Genet Mol Biol. 2020 May 29;43(2):e20190161. doi: 10.1590/1678-4685-GMB-2019-0161 (PMC7263422; doi:10.1590/1678-4685-GMB-2019-0161)
Supplement: Supplementary file 6 [file 1415-4757-GMB-43-2-e20190161-s3.pdf]

Supplementary Material to "Complete chloroplast genome sequence of *Caryocar brasiliense* Camb. (Caryocaraceae) and comparative analysis brings new insights into the plastome evolution of Malpighiales"

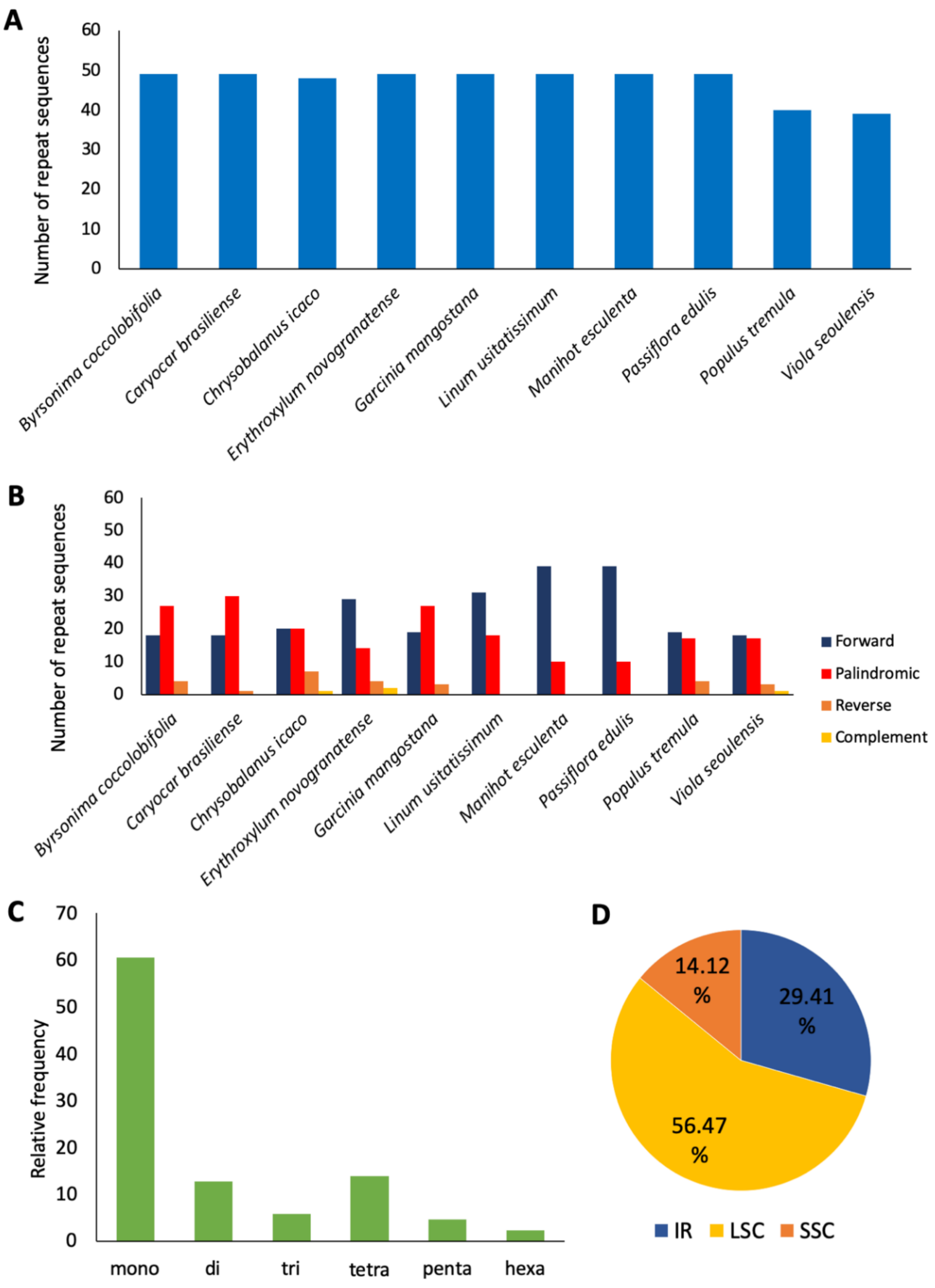

Figure S3 - Repeat and comparative analysis in *Caryocar brasiliense* chloroplast genome. A) Comparative total number of repeat sequences among Malpighiales chloroplast genome species; B) Comparative repeat types among Malpighiales chloroplast genome species; C) Frequency of microsatellites motifs in *C. brasiliense* chloroplast genome and D) Distribution of microsatellites in *C. brasiliense* chloroplast genome.
