# Supplementary material for: Complete chloroplast genome sequence of Caryocar brasiliense Camb. (Caryocaraceae) and comparative analysis brings new insights into the plastome evolution of Malpighiales
Source: Genet Mol Biol. 2020 May 29;43(2):e20190161. doi: 10.1590/1678-4685-GMB-2019-0161 (PMC7263422; doi:10.1590/1678-4685-GMB-2019-0161)
Supplement: Supplementary file 7 [file 1415-4757-GMB-43-2-e20190161-s6.pdf]

Genetics and Molecular Biology 43, 2 (2020)

Supplementary Material to "Complete chloroplast genome sequence of *Caryocar brasiliense* Camb. (Caryocaraceae) and comparative analysis brings new insights into the plastome evolution of Malpighiales"

Table S3 - Frequency of types of simple sequence repeats based on its motif length in *Caryocar brasiliense* chloroplast genome.

**Repeat unit**	**frequency**	**%**
mono	52	60,47
di	11	12,79
tri	5	5,81
tetra	12	13,95
penta	4	4,65
hexa	2	2,33
Total	86	100,00
