# Supplementary material for: Complete chloroplast genome sequence of Caryocar brasiliense Camb. (Caryocaraceae) and comparative analysis brings new insights into the plastome evolution of Malpighiales
Source: Genet Mol Biol. 2020 May 29;43(2):e20190161. doi: 10.1590/1678-4685-GMB-2019-0161 (PMC7263422; doi:10.1590/1678-4685-GMB-2019-0161)
Supplement: Supplementary file 8 [file 1415-4757-GMB-43-2-e20190161-s7.pdf]

Supplementary Material to "Complete chloroplast genome sequence of *Caryocar brasiliense* Camb. (Caryocaraceae) and comparative analysis brings new insights into the plastome evolution of Malpighiales"

Table S4 - Simple sequence repeats identified in *Caryocar brasiliense* chloroplast genome sequence.

Number	Consensus	Rep. Size	Iterations	Tract-size	Start	End	Region
1	TTGAA	5	3	15	833	847	LSC
2	A	1	13	13	1389	1401	LSC
3	A	1	11	11	1901	1911	LSC
4	AGAA	4	3	12	7270	7281	LSC
5	TTTA	4	3	12	10049	10060	LSC
6	A	1	14	14	10533	10546	LSC
7	A	1	10	10	10647	10656	LSC
8	T	1	11	11	10996	11006	LSC
9	A	1	10	10	11648	11657	LSC
10	A	1	10	10	11689	11698	LSC
11	AT	2	5	10	14406	14415	LSC
12	T	1	10	10	17884	17893	LSC
13	A	1	10	10	21267	21276	LSC
14	TAT	3	4	12	22775	22786	LSC
15	TAA	3	5	15	22859	22873	LSC
16	CAAA	4	3	12	31910	31921	LSC
17	A	1	10	10	32444	32453	LSC
18	T	1	10	10	35206	35215	LSC
19	AT	2	5	10	35262	35271	LSC
20	AT	2	6	12	37507	37518	LSC
21	AT	2	5	10	37580	37589	LSC
22	T	1	10	10	38485	38494	LSC
23	TA	2	5	10	39088	39097	LSC
24	T	1	10	10	41181	41190	LSC
25	A	1	12	12	41192	41203	LSC
26	T	1	10	10	41908	41917	LSC
27	TA	2	5	10	43796	43805	LSC
28	T	1	13	13	48386	48398	LSC
29	AT	2	5	10	49768	49777	LSC
30	A	1	10	10	52433	52442	LSC
31	A	1	10	10	52816	52825	LSC
32	T	1	10	10	56118	56127	LSC
33	A	1	10	10	57615	57624	LSC
34	C	1	13	13	57638	57650	LSC
35	A	1	10	10	60015	60024	LSC
36	T	1	10	10	60583	60592	LSC
37	ACAA	4	3	12	60676	60687	LSC

Number	Consensus	Rep. Size	Iterations	Tract-size	Start	End	Region
38	T	1	10	10	60921	60930	LSC
39	ATA	3	4	12	68680	68691	LSC
40	TCTT	4	3	12	75435	75446	LSC
41	A	1	10	10	76659	76668	LSC
42	A	1	11	11	76808	76818	LSC
43	T	1	10	10	77087	77096	LSC
44	T	1	12	12	79216	79227	LSC
45	TCAAT	5	3	15	79258	79272	LSC
46	AGAA	4	3	12	79945	79956	LSC
47	A	1	11	11	81636	81646	LSC
48	T	1	10	10	83850	83859	LSC
49	T	1	16	16	86456	86471	IR
50	AT	2	5	10	96737	96746	IR
51	T	1	12	12	101288	101299	IR
52	TATT	4	3	12	101348	101359	IR
53	AATGGA	6	3	18	102377	102394	IR
54	A	1	11	11	110421	110431	IR
55	AG	2	5	10	110512	110521	IR
56	A	1	10	10	114492	114501	IR
57	A	1	10	10	114712	114721	IR
58	A	1	10	10	115777	115786	IR
59	A	1	10	10	116260	116269	IR
60	T	1	10	10	117616	117625	IR
61	TAT	3	4	12	117873	117884	SSC
62	TTTTA	5	3	15	120631	120645	SSC
63	TCTT	4	3	12	120732	120743	SSC
64	TGGT	4	3	12	122433	122444	SSC
65	A	1	11	11	125343	125353	SSC
66	TATT	4	3	12	125730	125741	SSC
67	A	1	10	10	125954	125963	SSC
68	TTAA	4	3	12	127452	127463	SSC
69	A	1	11	11	127629	127639	SSC
70	A	1	10	10	128177	128186	SSC
71	T	1	10	10	128219	128228	SSC
72	T	1	10	10	128374	128383	SSC
73	AAT	3	4	12	132046	132057	IR
74	A	1	10	10	132306	132315	IR
75	T	1	10	10	133662	133671	IR
76	T	1	10	10	134145	134154	IR
77	T	1	10	10	135210	135219	IR
78	T	1	10	10	135430	135439	IR
79	CT	2	5	10	139410	139419	IR
80	T	1	11	11	139500	139510	IR
81	TCCATT	6	3	18	147537	147554	IR
82	ATAA	4	3	12	148573	148584	IR

Number	Consensus	Rep. Size	Iterations	Tract-size	Start	End	Region
83	A	1	12	12	148632	148643	IR
84	AT	2	5	10	153185	153194	IR
85	A	1	16	16	163460	163475	IR
