# Supplementary material for: Complete chloroplast genome sequence of Caryocar brasiliense Camb. (Caryocaraceae) and comparative analysis brings new insights into the plastome evolution of Malpighiales
Source: Genet Mol Biol. 2020 May 29;43(2):e20190161. doi: 10.1590/1678-4685-GMB-2019-0161 (PMC7263422; doi:10.1590/1678-4685-GMB-2019-0161)
Supplement: Supplementary file 9 [file 1415-4757-GMB-43-2-e20190161-s8.pdf]

Supplementary Material to "Complete chloroplast genome sequence of *Caryocar brasiliense* Camb. (Caryocaraceae) and comparative analysis brings new insights into the plastome evolution of Malpighiales"

Table S5 - Species list used in comparative analysis of Malpighiales order chloroplast genomes.

**Species**	**Family**	**Reference**	**NCBI**
Caryocar brasiliense	Caryocaraceae	This work	-
Garcinia mangostana	Clusiaceae	Jo et al., 2017	NC_036341.1
Chrysobalanus icaco	Chrysobalanaceae	Malé et al., 2014	NC_024061.1
Erythroxylum novogranatense	Erythroxylaceae	Unpublished	NC_030601.1
Manihot esculenta	Euphorbiaceae	Daniell et al., 2008	NC_010433.1
Linum usitatissimum	Linaceae	Lopes et al., 2017	NC_036356.1
Byrsonima coccolobifolia	Malpighiaceae	Menezes et al., 2018	NC_037191.1
Passiflora edulis	Passifloraceae	Cauz-Santos et al., 2017	NC_034285.1
Populus tremula	Salicaceae	Kersten et pal., 2016	NC_027425.1
Viola seoulensis	Violaceae	Cheon et al., 2015	NC_026986.1
