# Supplementary material for: Complete chloroplast genome sequence of Caryocar brasiliense Camb. (Caryocaraceae) and comparative analysis brings new insights into the plastome evolution of Malpighiales
Source: Genet Mol Biol. 2020 May 29;43(2):e20190161. doi: 10.1590/1678-4685-GMB-2019-0161 (PMC7263422; doi:10.1590/1678-4685-GMB-2019-0161)
Supplement: Supplementary file 10 [file 1415-4757-GMB-43-2-e20190161-s9.pdf]

Supplementary Material to "Complete chloroplast genome sequence of *Caryocar brasiliense* Camb. (Caryocaraceae) and comparative analysis brings new insights into the plastome evolution of Malpighiales"

Table S6 - Species list used in phylogenetic analysis of Malpighiales order.

ID	Family	Species	Code	Reference
1	Caryocaraceae	*Caryocar brasiliense*	Cbr	This work
2	Caryocaraceae	*Anthodiscus peruanus*	Ape	Xi et al., 2012
3	Clusiaceae	*Garcinia mangostana*	Gma	NC_036341.1
4	Chrysobalanaceae	*Acioa guianensis*	Agu	NC_030534.1
5	Chrysobalanaceae	*Afrolicania elaeosperma*	Ael	NC_030544.1
6	Chrysobalanaceae	*Angelesia splendens*	Asp	NC_030545.1
7	Chrysobalanaceae	*Atuna racemosa*	Ara	NC_030546.1
8	Chrysobalanaceae	*Chrysobalanus icaco*	Cic	NC_024061.1
9	Chrysobalanaceae	*Couepia caryophylloides*	Cca	NC_030547.1
10	Chrysobalanaceae	*Dactyladenia bellayana*	Dbe	NC_030555.1
11	Chrysobalanaceae	*Exellodendron barbatum*	Eba	NC_030558.1
12	Chrysobalanaceae	*Gaulettia elata*	Gel	NC_030559.1
13	Chrysobalanaceae	*Grangeria borbonica*	Gbo	NC_030560.1
14	Chrysobalanaceae	*Hirtella macrosepala*	Hma	NC_030561.1
15	Chrysobalanaceae	*Hunga gerontogea*	Hge	NC_030564.1
16	Chrysobalanaceae	*Kostermanthus robustus*	Kro	NC_030565.1
17	Chrysobalanaceae	*Licania alba*	Lal	NC_024064.1
18	Chrysobalanaceae	*Magnistipula butayei*	Mbu	NC_030576.1
19	Chrysobalanaceae	*Maranthes gabunensis*	Mga	NC_030577.1
20	Chrysobalanaceae	*Neocarya macrophylla*	Nma	NC_030580.1
21	Chrysobalanaceae	*Parastemon urophyllus*	Pur	NC_030517.1
22	Chrysobalanaceae	*Parinari campestris*	Pca	NC_024067.1
23	Erythroxylaceae	*Erythroxylum novogranatense*	Eno	NC_030601.1
24	Euphorbiaceae	*Euphorbia esula*	Ees	NC_033910.1
25	Euphorbiaceae	*Hevea brasiliensis*	Hbr	NC_015308.1
26	Euphorbiaceae	*Jatropha curcas*	Jcu	NC_012224.1
27	Euphorbiaceae	*Manihot esculenta*	Mes	NC_010433.1
28	Euphorbiaceae	*Ricinus communis*	Rco	NC_016736.1
29	Euphorbiaceae	*Vernicia fordii*	Vfo	NC_034803.1
30	Linaceae	*Linum usitatissimum*	Lus	NC_036356.1
31	Malpighiaceae	*Banisteriopsis caapi*	Bca	NC_037945.1
32	Malpighiaceae	*Byrsonima coccolobifolia*	Bco	NC_037191.1
33	Malpighiaceae	*Byrsonima crassifolia*	Bcr	NC_037192.1
34	Passifloraceae	*Passiflora cincinnata*	Pci	NC_037690.1
35	Passifloraceae	*Passiflora edulis*	Ped	NC_034285.1

ID	Family	Species	Code	Reference
36	Salicaceae	*Flacourtia indica*	Fin	NC_037410.1
37	Salicaceae	*Idesia polycarpa*	Ipo	NC_032060.1
38	Salicaceae	*Itoa orientalis*	Ior	NC_037411.1
39	Salicaceae	*Poliothyrsis sinensis*	Psi	NC_037412.1
40	Salicaceae	*Populus alba*	Pal	NC_008235.1
41	Salicaceae	*Populus angustifolia*	Pan	NC_037413.1
42	Salicaceae	*Populus tremula*	Pte	NC_027425.1
43	Salicaceae	*Populus nigra*	Pni	NC_037416.1
44	Salicaceae	*Populus trichocarpa*	Ptr	NC_009143.1
45	Salicaceae	*Salix arbutifolia*	Sar	NC_036718.1
46	Salicaceae	*Salix babylonica*	Sba	NC_028350.1
47	Salicaceae	*Salix magnifica*	Sma	NC_037424.1
48	Salicaceae	*Salix suchowensis*	Ssu	NC_026462.1
49	Salicaceae	*Salix tetrasperma*	Ste	NC_035744.1
50	Violaceae	*Viola seoulensis*	Vse	NC_026986.1
51	Vitaceae	*Vitis vinifera*	Vvi	NC_007957.1
52	Putranjivaceae	*Putranjiva roxburghii*	Pro	Xi et al., 2012
